# Association of Prenatal Dietary Toxicants and Inorganic Arsenic Exposure with Children’s Emotional and Behavioral Problems: ECLIPSES Study

**DOI:** 10.3390/toxics12060398

**Published:** 2024-05-29

**Authors:** Xiruo Kou, Josefa Canals, Monica Bulló, Nerea Becerra-Tomás, Cristina Jardí, Victoria Arija

**Affiliations:** 1 Nutrition and Mental Health (NUTRISAM) Research Group, Universitat Rovira i Virgili, 43204 Reus, Spainjosefa.canals@urv.cat (J.C.); nerea.becerra@urv.cat (N.B.-T.); cristina.jardi@urv.cat (C.J.); 2 Institut d’Investigació Sanitaria Pere Virgili (IISPV), 43204 Reus, Spain; monica.bullo@urv.cat; 3 Centre de Recerca en Avaluació i Mesura de la Conducta (CRAMC), Department of Psychology, Universitat Rovira i Virgili, 43007 Tarragona, Spain; 4 University Research Institute on Sustainablility, Climate Change and Energy Transition (IU-RESCAT), Universitat Rovira i Virgili, 43003 Tarragona, Spain; 5 CIBER Physiology of Obesity and Nutrition (CIBEROBN), Carlos III Health Institute, 28029 Madrid, Spain; 6 Center of Environmental, Food and Toxicological Technology—TecnATox, Rovira i Virgili University, 43201 Reus, Spain; 7 Nutrition and Metabolic Health Research Group, Department of Biochemistry and Biotechnology, Rovira i Virgili University, 43201 Reus, Spain; 8 Collaborative Research Group on Lifestyles, Nutrition and Smoking (CENIT), Tarragona-Reus Research Support Unit, Jordi Gol Primary Care Research Institute, 43003 Tarragona, Spain

**Keywords:** prenatal dietary toxicants, inorganic arsenic, neurodevelopment, behavioral development, food

## Abstract

Prenatal exposure to dietary toxicants is linked to neurocognitive issues, but its effect on early emotional and behavioral development in children is less clear. To explore the relationship between prenatal intake of As, iAs, Cd, MeHg, Pb, PCDD/Fs, DL-PCBs, and NDL-PCBs and emotional and behavioral issues in four-year-old children. This study included 192 mother–child pairs from the ECLIPSES study, assessing prenatal dietary toxicant exposure through a food-frequency questionnaire and Catalan Food Safety Agency data. Children’s emotional and behavioral scores were evaluated using the Child Behavior Checklist for ages 1.5–5 years. Multivariable regression and logistic models were used, focusing on iAs after finding significant preliminary associations. Increased prenatal dietary intake of iAs was associated with internalizing, externalizing, and attention-deficit/hyperactivity problems. Higher iAs levels (>4.16 μg/day) significantly increased the risk of total problems (OR = 2.94) and specific issues like anxious/depressed (OR = 4.88), anxiety (OR = 3.27), and oppositional defiant problems (OR = 4.30). High iAs consumption correlated with the intake of meat, eggs, cereals, tubers, fruits, and pulses Prenatal dietary iAs exposure is associated with various emotional and behavioral problems in children. Monitoring and reducing iAs levels in food are crucial for public health.

## 1. Introduction

Neurodevelopment in the fetus is a critical phase that establishes the foundation for lifelong neurological function, encompassing the organization of neural cells, the formation of neural networks, and the establishment of synaptic connections [1]. However, this delicate process faces considerable risks from prenatal toxicant exposure. The developing fetal brain, highly susceptible due to ongoing physiological development and immature detoxification mechanisms, is directly impacted by these toxicants, which can cross the placental barrier [2,3,4]. Therefore, prenatal toxicant exposure plays a crucial role in offspring’s neurodevelopment, potentially influencing their overall health and well-being.

So far, many studies have illustrated that prenatal toxicant exposure is linked to impaired cognitive and motor development, including language, cognition, intelligence, etc. [5,6,7,8,9,10]. Nevertheless, the research on the association of prenatal toxicant exposure with emotional and behavioral development is limited and not fully consistent. For instance, some studies have presented that prenatal exposure to metals is associated with autism, emotional control, hyperactivity/inattention, and depression among children [11,12,13,14]. However, researchers have also found that the association between prenatal metal exposure and autism is null [15] and the associations of persistent organic pollutants (POPs) and heavy metals with behavioral problems are inconsistent [16].

Emotional and behavioral disorders among children are on the rise globally [17,18]. Prevalence rates for specific clinical conditions such as anxiety, depression, and attention-deficit/hyperactivity disorder (ADHD) in children are 6.5%, 2.6%, and 3.4%, respectively [19]. The occurrence of these disorders early in life may persist throughout an individual’s lifespan, exerting a stable adverse influence on their mental health trajectory [20]. Therefore, more research on identifying the associations of prenatal toxicant exposure and the emotional and behavioral development of children is essential in terms of both public health and clinical perspectives.

Dietary toxicants have emerged as a focal point in recent years [21,22,23]. In previous research, we identified prenatal daily arsenic (As) and dioxin-like polychlorinated biphenyl (DL-PCB) intake either exceeding or closely approaching the thresholds set by the European Food Safety Authority (EFSA) during the gestational period [24]. In this study, we investigate the associations of prenatal dietary toxicants, including As, inorganic As (iAs), cadmium (Cd), MeHg, lead (Pb), polychlorinated dibenzo-p-dioxins and dibenzofurans (PCDD/Fs), DL-PCBs, and non-DL-PCBs (NDL-PCBs) with emotional and behavioral development among four-year-old children in a Mediterranean city with bustling industries in Spain.

## 2. Methods

### 2.1. Design and Participant Selection

The analysis presented here was part of the ECLIPSES study, community-centered research focused on pregnant women in the Tarragona region of Spain. The subjects included in this subset were enlisted during their initial prenatal appointments at primary care facilities, facilitated by midwives. To be eligible for participation, individuals had to meet specific criteria: they had to be healthy women aged 18 or older, within the first 12 weeks of gestation, without anemia, and comprehending the local (Catalan) or official (Spanish) language spoken in the area. Exclusion criteria comprised a history of multiple pregnancies, the utilization of iron supplements during the early stages of pregnancy, a background of severe diseases with potential impacts on nutritional development (e.g., anemia, cancer, diabetes, malabsorption, or liver disease), and immunosuppression. More detailed information can be referenced elsewhere [25]. Between 2013 and 2017, a total of 791 pregnant women were enlisted during their first prenatal appointment, establishing a partnership with midwives across 12 sexual and reproductive healthcare facilities (ASSIR) linked with the Catalan Institute of Health in Tarragona province, Catalonia, Spain. All participants signed an informed consent form. The study complied with the tenets of the Declaration of Helsinki.

### 2.2. Gathering Maternal Information

Maternal obstetrical and sociodemographic information gathering: demographic information (including maternal age, economic status, and educational attainment), health behaviors (such as smoking and dietary habits), and anthropometric measurements throughout each trimester of pregnancy was gathered by midwives and nutritionists. Maternal weight and height were computed, and body mass index (BMI) was determined during the initial trimester. Information on social class was determined based on education and occupation as gathered with local categorization of occupations [26].

Lifestyle behaviors: Data on maternal lifestyle, encompassing smoking status and dietary patterns, were gathered. Dietary patterns were evaluated retrospectively using a validated 45-item food frequency questionnaire (FFQ), self-administered during 3 trimesters of gestation [27]. Dietitians conducted the analysis of the FFQ, calculating daily consumption of food, energy, and nutrient intake by applying the Spanish food-composition table [28,29]. To evaluate diet quality, a Mediterranean Diet (MedDiet) score was also administered [27]. The scoring method allocated increasing values of 0, 1, or 2 points depending on the consumption of various food items. Participants received scores ranging from 0 to 18, with elevated scores indicating improved dietary quality.

The maternal prenatal average toxicant intake through the three trimesters, encompassing As, iAs, Cd, MeHg, Pb, PCDD/Fs, DL-PCBs, and NDL-PCBs, was determined using the toxicant database of food from the Catalan Food Safety Agency, as detailed in a previously published paper [24]. Further methodological and results information is available in the publicly accessible Catalan Food Safety Agency report [30].

Psychological information: Maternal psychological distress was evaluated using the State-Trait Anxiety Inventory in both the first and third trimesters, and the scores were averaged to represent distress levels across the entire gestational period [31].

### 2.3. Children’s Data Collection

Birth Data Collection: Relevant information concerning the birth was sourced from hospital obstetric records, including details of the infant’s sex and gestational age (calculated based on the duration since the onset of the most recent menstrual cycle). Mothers were also questioned regarding their selected infant feeding methods.

Psychological data: Emotional and behavioral issues in children at the age of 4 years were assessed by parents utilizing the Child Behavior Checklist for ages 1.5–5 (CBCL 1.5–5) [32]. The CBCL 1.5–5 comprises 99 items, each offering three response options: “not true,” “somewhat or sometimes true,” and “very true or often true.” It encompassed six empirically based syndrome scales, including emotional reactivity, anxiety/depression, somatic complaints, withdrawal, attention problems, and aggressive behavior. The scales related to emotional reactivity, anxiety/depression, somatic complaints, and withdrawal collectively form the internalizing problems scale, while attention problems and aggressive behavior scales constitute the externalizing problems scale. The total problem scale encompassed all syndromic scales. Additionally, DSM 5-oriented scales, such as depressive problems, anxiety problems, autism spectrum problems, attention-deficit/hyperactivity problems, and oppositional defiant problems, were included. T-scores from the Spanish version were employed for all scales. In terms of Syndrome Scales and DSM-Oriented Scales, T scores between 65 and 69 are categorized as in the borderline range, and scores above 69 are categorized as in the clinical range. Regarding the Broad-band scales, scores of 60 and 63 delimited the borderline and clinical ranges, respectively, and scores below 60 were considered within the normal range [32]. For this study, we categorized the risk of any psychological problems when scores exceeded the borderline range. The internal consistency of the Spanish version demonstrated a range from moderate to good [33].

### 2.4. Statistical Analyses

Before conducting analyses, mean imputation was used to replace missing values in the MedDiet score during pregnancy (1.0%) and the State-Trait Anxiety Inventory score (3.1%) variables using the mean of the available non-missing cases [34]. Subsequently, after the missing values were replaced in these two variables, all the potential covariates of interest were completed. Descriptive statistics were presented with quantitative variables expressed as means and standard deviations, and qualitative variables as numbers and percentages. Based on the Kolmogorov–Smirnov test, prenatal dietary toxicants were found to follow a normal distribution. An analysis of variance (ANOVA) test and Chi-square test were applied to compare behavioral problem scales and the frequency of psychological problem risk among children across different tertiles of prenatal dietary toxicants. The toxicants with the lowest concentrations (tertile 1) served as the reference category. Multivariable regression models were employed to estimate the association between prenatal dietary iAs and CBCL 1.5–5 (continuous). Additionally, separate multivariate-adjusted logistic models were applied to estimate the association of different levels of prenatal dietary iAs intake and psychological problem risk. These models were a priori adjusted for covariates potentially influencing this association. Maternal covariates included age, BMI (categorized as normal weight, overweight, and obesity) [35], social class (categorized as low vs. middle/high), smoking status (categorized as never, ex/smoker), Mediterranean Diet adherence during pregnancy (continuous), energy intake during pregnancy (continuous), and State-trait anxiety inventory score (continuous). Children’s covariates included gestational age, sex, and type of feeding (breast vs. mixed/formula). A statistical power analysis was conducted, considering the sample data, with a significance level alpha of 0.05 and a medium effect size, where a power (1-β) of 0.96 was obtained. This suggested that the number of subjects was adequate to detect children’s emotional and behavioral problems in the sample. Statistical analyses were conducted by SPSS software (version 27.0 for Windows; SPSS Inc.), and figures were produced using R (version 4.3.2). All statistical significance was set at *p* < 0.05.

## 3. Results

### 3.1. Study Design

**Figure 1:**
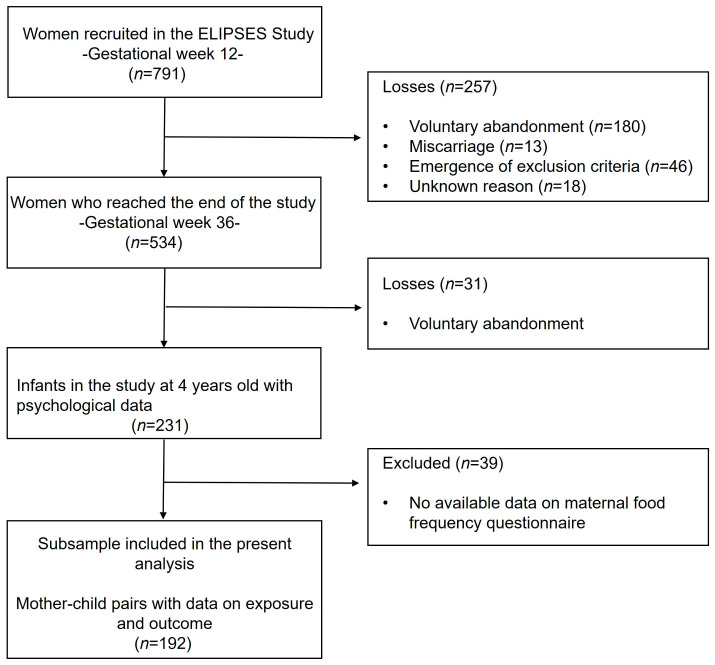
Flowchart of study.

Out of the initial cohort of women recruited (*n* = 791), 192 mother–child pairs with available information on both the prenatal food frequency questionnaire and behavioral assessments of children at four years old were taken into account in the current analyses (Figure 1).

### 3.2. Characteristics of Pregnant Women

The general characteristics of pregnant women are described based on their dietary intake of iAs in Table 1 and other toxicant levels in Supplementary Table S1. No differences were identified in age, BMI, socioeconomic level, smoking status, MedDiet score, or State-trait anxiety inventory score among the tertiles of dietary toxicant intake. A significant increase in total energy intake was found in the higher tertiles of dietary toxicant intake.

### 3.3. Psychological Data of Four-Year-Old Children

No significant differences were identified in the varying levels of maternal prenatal dietary intake of toxicants (As, Cd, MeHg, Pb, PCDD/Fs, DL-PCBs, and NDL-PCBs) and behavioral problem scales (Supplementary Table S2), with the exception of iAs. Maternal prenatal dietary intake of iAs in relation to the emotional and behavioral problem scale of four-year-old children is shown in Table 2. Within the syndrome scales, heightened iAs intake was associated with increased scales of somatic complaints, withdrawal, and attention problems (all *p* < 0.05). Consequently, increased iAs intake was also significantly associated with the internalizing and total problem scales. Regarding DSM-oriented scales, higher iAs intake was associated with higher attention-deficit/hyperactivity problems (all *p* < 0.05).

### 3.4. Association between Prenatal iAs Intake and Children’s Psychological Problem Scores

Table 3 shows the associations of maternal prenatal iAs intake and children’s emotional and behavioral problem scales. After controlling for potential confounding factors, the higher tertile of iAs was positively associated with being anxious/depressed (β = 3.18), withdrawn behavior (β = 4.41), attention problems (β = 3.59), aggressive behavior (β = 3.21), internalizing problems (β = 6.92), externalizing problems (β = 5.39), total problems (β = 6.77), DSM depressive problems (β = 3.25), DSM anxiety problems (β = 3.41), DSM autism spectrum problems (β = 2.88), DSM attention-deficit/hyperactivity problems (β = 3.59), and DSM oppositional defiant problems (β = 3.12) (all *p* < 0.05).

Additionally, when we treated the exposure variable as continuous, each 10 increment unit of iAs was positively associated with being anxious/depressed (β = 0.07), somatic complaints (β = 0.08), withdrawn behavior (β = 0.07), internalizing problems (β = 0.15), externalizing problems (β = 0.10), total problems (β = 0.14), DSM depressive problems (β = 0.09), DSM anxiety problems (β = 0.09), DSM attention-deficit/hyperactivity problems (β = 0.07), and DSM oppositional defiant problems (β = 0.07) (all *p* < 0.05).

### 3.5. Association between Prenatal iAs Intake and Risk of Children’s Psychological Problems

**Figure 2:**
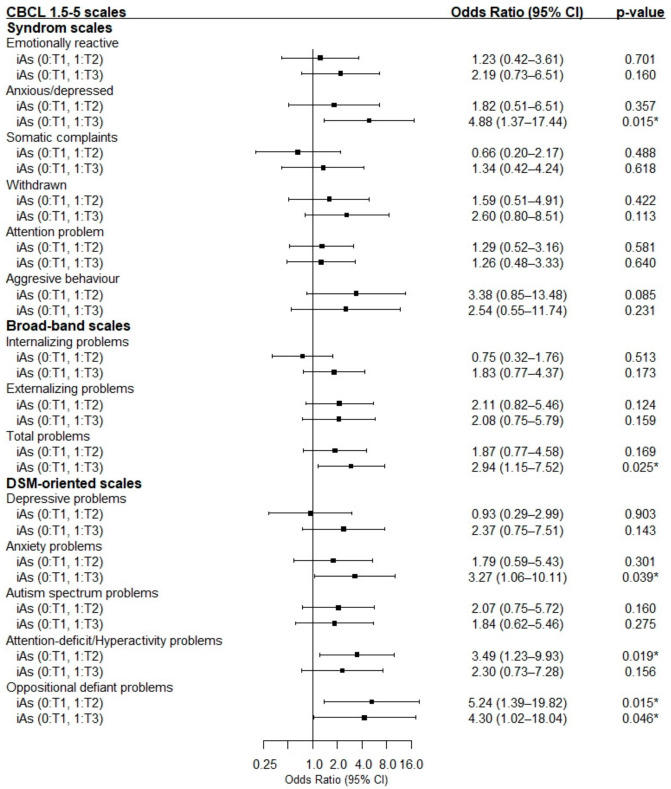
Association of different tertiles of prenatal iAs intake with children’s psychological problem risk. *, Results are statistically significant.

After controlling for potential confounding factors, the higher tertile of iAs was associated with an increased risk of anxious/depressed problems (odd ratio (OR) = 4.88), total problems (OR = 2.94), DSM anxiety problems (OR = 3.27), DSM attention-deficit/hyperactivity problems (OR = 3.49), and DSM oppositional defiant problems (OR = 4.30) (all *p* < 0.05) (Figure 2).

In addition, Table 4 illustrates that intake of meat, eggs, cereal, tubers, fruits, and pulses was positively associated with dietary iAs consumption (all *p* < 0.05).

## 4. Discussion

In the current birth cohort study performed in Tarragona, Spain, a region known for its heavy industrial activity, the association between the dietary intake of various toxicants among pregnant women with emotional and behavioral outcomes in their four-year-old children was examined. Among the all toxicants analyzed, only iAs was associated with several adverse emotional and behavioral scales, including anxiety, depression, attention problems, and autism spectrum disorder.

Exposure to prenatal toxicants has been linked to a range of adverse neurocognitive outcomes [5,6,10]. However, their relationship with early emotional and behavioral development remains less clear and requires further investigation. While mental disorders among children are on the rise globally, the current literature on the association of prenatal toxicant exposure with emotional and behavioral problems among children remains inconclusive [17,18]. For example, prenatal Pb concentration in whole blood was positively associated with children’s emotional problems [12]. Furthermore, elevated maternal urinary levels of cobalt, nickel, Cd, and Pb were positively associated with an increased risk of DSM-5-oriented issues related to anxiety, autism spectrum disorders, and oppositional defiant problems [14]. Moreover, elevated prenatal Cd levels in whole blood were linked to higher ADHD scores, specifically in girls [36]. Additionally, prenatal serum PCBs were positively associated with abnormal scores for hyperactivity [37]. In contrast, earlier research found no links between prenatal exposure of PCBs and emotional or behavioral problems among children [16,38]. Meanwhile, it also has been demonstrated that prenatal dietary exposure to dioxins during pregnancy is not associated ADHD symptoms [39]. In addition, all associations between prenatal metal levels (Pb, Hg, selenium, and manganese) and autism were either inconclusive or inconsistent [15]. In the current study, we found that among all prenatal dietary toxicants, high iAs was associated with elevated emotional and behavioral problems among four-year-old children, although higher dietary intake of iAs (4.16 μg/day) in this study was still relatively low compared to the tolerable daily intake as considered by the EFSA (0.3 μg/kg bw/day; given that the average weight in our population was 64.6 kg, this equated to 19.38 μg/day) [22].

Until now, the association between prenatal iAs exposure and children’s neurodevelopment has primarily been found in neurocognitive aspects, including intelligence and cognitive abilities [40,41,42,43]. Our findings emphasize the negative association of prenatal dietary iAs exposure with neurodevelopment and fill the research gap in understanding its association with behavioral development. The potential explanations of negative association could be that (a) iAs might be genotoxic; the development of the brain starts in early gestation and continues after birth, meaning prenatal iAs exposure might affect offspring neurodevelopment, consequently influencing behavioral development over time [44,45]; (b) iAs might be associated with diminished glial and neuronal populations, neural tube defects, reduced brain weight, and alterations in neurotransmitter systems [46,47,48]; (c) iAs might potentially enhance cellular apoptosis, prompt alterations in neurotransmitters, contribute to neurodegeneration, and induce peripheral neuropathy [49]; (d) iAs neurotoxicity might include oxidative stress, proinflammatory effects, mitochondrial dysfunction, and disruptions in synaptic transmission, all of which actively contribute to the impairment of behavior and overall neurological health [50].

Lately, iAs intake has become a focal point of discussion within the European Commission, driven by health concerns raised in EFSA’s recent risk assessment of this toxicant. [51]. When iAs concentrations in water are below 10 μg/L, the main route of exposure to iAs for the general population is predominantly through food sources [52]. In Spain, drinking water typically adheres to EU regulations governing iAs levels (with a reported median concentration below 1 μg/L); hence, diet is the primary contributor to iAs intake in our population [53,54]. As is known, cereals and cereal products, especially rice, are the main contributors of iAs intake in the Spanish population and other populations with similar gastronomic cultures [24,55]. In the current study, we found that cereal, tubers, eggs, pulses, red meat, and fruits were independently associated with elevated iAs intake among our population, which was partly in line with other studies [55,56]. In light of the aforementioned inverse association of prenatal dietary iAs with emotional and behavioral outcomes among children, it is imperative for local governments to conduct regular monitoring and inspection of food items contributing to higher iAs intake. Taking necessary measures to reduce iAs levels in these items is essential for safeguarding public health.

To our knowledge, this study is the first to investigate the association between different prenatal dietary toxicants and behavioral development in Spain. The study has several strengths: Firstly, the rich dataset from ECLIPSES enabled us to incorporate potential confounding factors that may influence children’s emotional and behavioral development. For instance, maternal prenatal mental status during pregnancy was taken into account, aiming to circumvent potential genetic influences. Secondly, the use of FFQs allowed us to utilize habitual dietary intake instead of food consumption over a few selected days, which is particularly useful for assessing toxicants, which accumulate in the body due to long-term dietary exposure. Finally, using toxicant data collected from locally sourced products provided convincing information for the local community. Nevertheless, there also some limitations that deserve comments: (1) The relatively wide confidence intervals for certain associations posed challenges in definitively determining the exact strength of the associations. (2) The relatively small sample size of the current study was a potential concern.

## 5. Conclusions

In summary, our study finds that, among all the prenatal dietary toxicants, iAs is negatively associated with emotional and behavioral development in four-year-old children. Therefore, regular monitoring and inspection of the foods that contribute to higher iAs intake, specific dietary recommendations for fertile and/or pregnant women, along with measures to reduce iAs in food are imperative for safeguarding childhood neurodevelopment.

## Figures and Tables

**Table 1:** General characteristics of pregnant women by tertile of dietary iAs intake.

		Dietary iAs Intake			
Characteristics	Total (*n* = 192)	Tertile 1 <3.04 μg/day (*n* = 64)	Tertile 2 3.04–4.16 μg/day (*n* = 64)	Tertile 3 >4.16 μg/day (*n* = 64)	
Maternal Characteristics	Summary Statistics				*p*
Age (years), mean ± SD	31.88 ± 4.44	32.45± 4.17	31.72 ± 4.67	31.47 ± 4.50	0.430
BMI (kg/m^2^), n (%)					0.404
<25 (normal weight)	110 (57.3%)	33 (51.6%)	39 (60.9%)	38 (59.4%)	
25–29 (overweight)	56 (29.2%)	18 (28.1%)	18 (28.1%)	20 (31.3%)	
≥30 (obesity)	26 (13.5%)	13 (20.3%)	7 (11.0%)	6 (9.3%)	
Social class, n (%)					0.148
Low	16 (8.3%)	2 (3.1%)	6 (9.4%)	8 (12.5%)	
Middle/High	176 (91.7%)	62 (96.9%)	58 (90.6%)	56 (87.5%)	
Smoking status, n (%)					0.930
Never	132 (68.8%)	44 (68.8%)	43 (67.2%)	45 (70.3%)	
Ex-smoker/Smoker	60 (31.3%)	20 (31.3%)	21 (32.8%)	19 (29.7%)	
MedDiet during pregnancy (score), mean ± SD	9.83 ± 2.45	10.02 ± 2.48	9.53 ± 2.27	9.92± 2.59	0.495
Energy intake during pregnancy (kcal/d), mean ± SD	1956.17 ± 521.15	**1696.30 ± 390.73 ^ac^**	**1972.12 ± 442.64 ^ab^**	**2200.10 ± 587.79 ^bc^**	**<0.001**
State-trait anxiety inventory score, mean ± SD	14.80 ± 6.80	14.58 ± 7.35	14.76 ± 5.94	15.07 ± 7.13	0.920

*p*-value for comparisons between categories was calculated by Pearson’s chi-square test or one-factor ANOVA tests. a, b, c: <0.05, conducted by ANOVA with Bonferroni post hoc test. Abbreviations: MedDiet, Mediterranean diet; BMI, early pregnancy Body Mass Index; iAs, inorganic arsenic. Results in bold are statistically significant.

**Table 2:** Mean scores and frequency of risk of psychological problems according to tertiles of prenatal dietary iAs intake.

	Dietary iAs Intake						
Emotional and Behavior Scale	Tertile 1 <3.04 μg/day		Tertile 2 3.04–4.16 μg/day		Tertile 3 >4.16 μg/day		*p*
	Mean	SD	Mean	SD	Mean	SD	
**Syndrome Scales**							
Emotionally reactive	56.33	9.14	56.28	8.21	58.73	9.66	0.216
% *	15.6%		15.6%		21.9%		0.564
Anxious/depressed	54.73	7.04	56.06	7.59	57.59	7.73	0.098
% *	**7.8%**		**12.5%**		**23.4%**		**0.037**
Somatic complaints	**54.91**	**6.95**	**54.14**	**5.75**	**56.95**	**7.11**	**0.048 ^b^**
% *	12.5%		10.9%		20.3%		0.274
Withdrawn	**55.95 ^a^**	**6.79**	**57.63**	**7.05**	**59.92 ^a^**	**8.13**	**0.010**
% *	12.5%		17.2%		20.3%		0.490
Attention problems	**55.97 ^a^**	**6.69**	**58.23**	**7.84**	**59.08 ^a^**	**7.13**	**0.045**
% *	21.9%		26.6%		26.6%		0.779
Aggressive behavior	54.02	5.53	55.97	7.92	56.66	8.66	0.120
% *	6.3%		15.6%		12.5%		0.238
**Broad-band scales**							
Internalizing problems	**52.27 ^a^**	**12.38**	**53.55**	**11.99**	**58.27 ^a^**	**11.00**	**0.011**
% *	34.4%		28.1%		43.8%		0.177
Externalizing problems	51.36	9.31	53.25	12.69	55.66	11.10	0.093
% *	18.8%		29.7%		26.6%		0.338
Total problems	**51.98 ^a^**	**11.17**	**53.61**	**13.21**	**57.61 ^a^**	**11.81**	**0.027**
% *	21.9%		29.7%		35.9%		0.215
**DSM-Oriented Scales**							
Depressive problems	55.27	6.70	55.98	7.03	58.22	7.42	0.050
% *	14.1%		12.5%		21.9%		0.303
Anxiety problems	55.86	7.42	57.25	8.11	59.25	8.62	0.060
% *	10.9%		17.2%		26.6%		0.070
Autism spectrum problems	56.19	6.83	57.11	7.27	58.55	7.38	0.174
% *	15.6%		23.4%		21.9%		0.509
Attention-deficit/Hyperactivity problems	**55.16**	**6.14**	**58.36**	**8.75**	**58.38**	**8.39**	**0.030 ^b^**
% *	10.9%		28.1%		21.9%		0.050
Oppositional defiant problems	53.59	5.28	55.63	7.74	55.80	7.45	0.137
% *	6.3%		18.8%		14.1%		0.105

* frequency of risk of psychological problems according to iAs tertiles. *p*-value for comparisons among categories was calculated by one-factor ANOVA tests and Chi-square test. a: *p* < 0.05, conducted by Bonferroni post hoc test. b: There was no difference among iAs tertiles in Bonferroni post hoc test. Abbreviations: iAs, inorganic arsenic. Results in bold are statistically significant.

**Table 3:** Association between children’s psychological problems and prenatal dietary iAs intake.

	Dietary iAs Intake									
	Tertile 1 (Ref.)	Tertile 2			Tertile 3			Per 10 Increment Unit		
		β	95%CI	*p*	β	95%CI	*p*	β	95%CI	*p*
**Syndrome scale**										
**Emotionally reactive**										
Unadjusted model		−0.05	(−3.19–3.10)	0.977	2.41	(−0.07–5.55)	0.133	0.07	(−0.01–0.14)	0.078
Adjusted model		0.44	(−2.70–3.59)	0.781	3.30	(−0.03–6.64)	0.052	0.08	(0.00–0.15)	0.056
**Anxious/depressed**										
Unadjusted model		1.33	(−1.27–3.93)	0.315	**2.86**	**(0.26–5.46)**	**0.031**	**0.06**	**(0.00–0.12)**	**0.047**
Adjusted model		1.68	(−0.92–4.27)	0.204	**3.18**	**(0.44–5.93)**	**0.023**	**0.07**	**(0.00–0.13)**	**0.036**
**Somatic complaints**										
Unadjusted model		−0.77	(−3.08–1.55)	0.514	2.05	(−0.26–4.36)	0.082	**0.08**	**(0.03–0.14)**	**0.002**
Adjusted model		−1.12	(−3.45–1.22)	0.347	1.54	(−0.93–4.02)	0.219	**0.08**	**(0.02–0.14)**	**0.007**
**Withdrawn**										
Unadjusted model		1.67	(−0.89–4.23)	0.200	**3.97**	**(1.41–6.53)**	**0.003**	**0.06**	**(0.00–0.12)**	**0.043**
Adjusted model		1.86	(−0.75–4.46	0.161	**4.41**	**(1.65–7.18)**	**0.002**	**0.07**	**(0.00–0.13)**	**0.038**
**Attention problems**										
Unadjusted model		2.27	(−0.26–4.79)	0.078	**3.11**	**(0.59–5.63)**	**0.016**	0.05	(0.00–0.12)	0.067
Adjusted model		**2.64**	**(0.15–5.13)**	**0.037**	**3.59**	**(0.95–6.22)**	**0.008**	0.06	(0.00–0.12)	0.059
**Aggressive behavior**										
Unadjusted model		1.95	(−0.66–4.57)	0.142	**2.64**	**(0.03–5.25)**	**0.048**	0.05	(−0.01–0.11)	0.121
Adjusted model		2.37	(−0.18–4.92)	0.068	**3.21**	**(0.51–5.92)**	**0.020**	0.06	(−0.01–0.12)	0.088
**Broad-band scales**										
**Internalizing problems**										
Unadjusted model		1.28	(−2.84–5.40)	0.540	**6.00**	**(1.88–10.12)**	**0.005**	**0.14**	**(0.04–0.23)**	**0.006**
Adjusted model		1.89	(−2.29–6.07)	0.374	**6.92**	**(2.48–11.35)**	**0.002**	**0.15**	**(0.04–0.25)**	**0.006**
**Externalizing problems**										
Unadjusted model		1.89	(−1.99–5.77)	0.337	**4.30**	**(0.42–8.17)**	**0.030**	0.09	(0.00–0.18)	0.055
Adjusted model		2.79	(−1.03–6.60)	0.151	**5.39**	**(1.35–9.44)**	**0.009**	**0.10**	**(0.00–0.19)**	**0.041**
**Total problems**										
Unadjusted model		1.62	(−2.59–5.84)	0.448	**5.62**	**(1.41–9.84)**	**0.009**	**0.13**	**(0.03–0.23)**	**0.012**
Adjusted model		2.42	(−1.75–6.59)	0.253	**6.77**	**(2.35–11.19)**	**0.003**	**0.14**	**(0.04–0.24)**	**0.007**
**DSM-Oriented Scales**										
**DSM Depressive problems**										
Unadjusted model		0.72	(−1.74–3.18)	0.565	**2.95**	**(0.49–5.41)**	**0.019**	**0.08**	**0.03–0.14**	**0.005**
Adjusted model		0.92	(−1.56–3.41)	0.465	**3.25**	**(0.62–5.89)**	**0.016**	**0.09**	**0.03–0.15**	**0.005**
**DSM Anxiety problems**										
Unadjusted model		1.39	(−1.42–4.20)	0.331	**3.39**	**(0.58–6.20)**	**0.018**	**0.08**	**0.01–0.14**	**0.022**
Adjusted model		1.48	(−1.33–4.30)	0.300	**3.41**	**(0.42–6.39)**	**0.026**	**0.09**	**0.01–0.15**	**0.018**
**DSM Autism spectrum problems**										
Unadjusted model		0.92	(−1.58–3.42)	0.468	2.36	(−0.14–4.86)	0.064	0.04	−0.02–0.10	0.148
Adjusted model		1.37	(−1.11–3.86)	0.277	**2.88**	**(0.25–5.52)**	**0.032**	0.05	−0.02–0.11	0.141
**DSM Attention-deficit/Hyperactivity problems**										
Unadjusted model		**3.20**	**(0.47–5.94)**	**0.022**	**3.22**	**(0.48–5.95)**	**0.021**	0.06	0.00–0.13	0.055
Adjusted model		**3.31**	**(0.59–6.03)**	**0.017**	**3.59**	**(0.70–6.48)**	**0.015**	**0.07**	**0.00–0.14**	**0.046**
**DSM Oppositional defiant problems**										
Unadjusted model		2.03	(−0.38–4.44)	0.098	2.20	(−0.21–4.61)	0.073	0.05	0.00–0.11	0.062
Adjusted model		**2.56**	**(0.11–5.01)**	**0.040**	**3.12**	**(0.52–5.72)**	**0.019**	**0.07**	**0.01–0.13**	**0.028**

Model conducted by multiple linear regression. Model adjusted by age, BMI (low, middle, high), social class (low vs. middle/high), smoking status (never, ex/smoker), MedDiet during pregnancy (score), energy intake during pregnancy, intervention group, State-trait anxiety inventory score, gestational age at birth, child sex, type of feeding (breast vs. mix/formula). Abbreviations: iAs, inorganic arsenic; ref., reference; CI, confidence interval. Results in bold are statistically significant.

**Table 4:** Association of maternal characteristics and dietary intake of iAs.

	Dietary iAs Intake		
Maternal Characteristics	β *	(95% CI)	*p*
Age (years)	0.11	(−0.26–0.49)	0.554
BMI (kg/m^2^)			
<25 (normal weight) (ref.)			
25–29 (overweight)	1.06	(−2.55–4.68)	0.562
≥30 (obesity)	4.19	(−0.55–8.94)	0.083
Social class			
Low (ref.)			
Middle/High	0.50	(−5.19–6.20)	0.861
Smoking status			
Never (ref.)			
Ex-smoker/Smoker	−0.39	(−3.86–3.08)	0.825
MedDiet during pregnancy (score)	−0.64	(−1.54–0.25)	0.156
Iron supplement (mg/day)	0.02	(−0.05–0.09)	0.552
State-trait anxiety inventory score	−0.03	(−0.25–0.19)	0.796
Milk	0.01	(0.00–0.02)	0.179
Cheese	−0.12	(−0.28–0.04)	0.148
White/processed meat	−0.01	(−0.09–0.06)	0.691
Red meat	**0.14**	**(0.02–0.25)**	**0.019**
White fish	0.02	(−0.13–0.17)	0.772
Blue fish	−0.12	(−0.25–0.01)	0.067
Seafood	0.29	(−0.07–0.65)	0.117
Eggs	**0.30**	(0.11–0.48)	**0.002**
Sweet cereal	−0.05	(−0.12–0.03)	0.204
Cereal and tubers	**0.33**	**(0.28–0.39)**	**<0.001**
Vegetables	−0.03	(−0.08–0.03)	0.319
Fruits	**0.03**	**(0.01–0.05)**	**0.001**
Pulses	**0.27**	**(0.05–0.48)**	**0.015**

Model conducted by multivariate linear regression. * Mean iAs intake was 3.96 μg/day; to understand the β more obviously, we applied a 10 unit change for iAs. Abbreviations: MedDiet, Mediterranean diet; BMI, early pregnancy Body Mass Index; iAs, inorganic arsenic. Results in bold are statistically significant.

## Data Availability

Data available on request from authors.

## Supplementary Materials

The following supporting information can be downloaded at: https://www.mdpi.com/article/10.3390/toxics12060398/s1.
