# Supplementary material for: Association of Prenatal Dietary Toxicants and Inorganic Arsenic Exposure with Children’s Emotional and Behavioral Problems: ECLIPSES Study
Source: Toxics. 2024 May 29;12(6):398. doi: 10.3390/toxics12060398 (PMC11209564; doi:10.3390/toxics12060398)
Supplement: Supplementary file 1 [file toxics-12-00398-s001.zip › toxics-3023752-supplementary.pdf]

Supplementary Table 1. General characteristics of pregnant women and offspring according to tertiles of toxicant consumption

**Characteristics**	**Dietary toxicants**																				
	As			Cd			MeHg			Pb			PCDD/Fs			DL-PCBs			NDL-PCBs		
	T1 <194.83 µg/d	T3 >298.09 µg/d	P	T1 <5.11 µg/d	T3 >6.83 µg/d	P	T1 <3.62 µg/d	T3 >5.51 µg/d	P	T1 <2.92 µg/d	T3 >3.60 µg/d	P	T1 <8.55 TEQ/d	T3 >10.90 TEQ/d	P	T1 <14.00 TEQ/d	T3 >20.55 TEQ/d	P	T1 <133.91 TEQ/d	T3 >201.92 TEQ/d	P
**Maternal characteristics**																					
Age (years)	31.17±4.82	32.86±3.75	0.083	31.98±4.30	31.91±4.57	0.955	31.34±4.83	32.97±4.27	0.055	31.84±4.19	32.45±4.56	0.370	31.53±4.33	32.50±4.34	0.393	31.39±5.05	32.81±4.38	0.121	31.19±4.84	32.75±4.36	0.128
BMI (kg/m2), %			0.271			0.853			0.521			0.998			0.454			0.276			0.507
<25 (normal weight)	59.4	59.4		60.9	53.1		62.5	53.1		56.3	57.8		53.1	62.5		64.1	50.0		59.4	51.6	
25-29 (overweight)	21.9	28.1		28.1	32.8		21.9	35.9		29.7	28.1		28.1	25.0		21.9	39.1		23.4	35.9	
≥30 (obesity)	18.7	12.5		11.0	14.1		15.6	11.0		14.0	14.1		18.8	12.5		14.0	10.9		17.2	12.5	
Social class, %			0.620			0.339			0.620			0.182			0.761			0.620			0.620
Low	6.2	10.9		9.4	14.1		7.8	10.9		3.1	10.9		6.3	9.4		7.8	10.9		7.8	10.9	
Middle/High	93.8	89.1		90.6	85.9		92.2	89.1		96.9	89.1		93.7	90.6		92.2	89.1		92.2	89.1	
Smoking status, %			0.930			0.068			0.389			0.389			0.105			0.131			0.930
Never	67.2	68.8		75.0	57.8		70.3	73.4		73.4	62.5		70.3	59.4		75.0	71.9		70.3	68.7	
Ex-smoker/Smoker	32.8	31.2		25.0	42.2		29.7	26.6		26.6	37.5		29.7	40.6		25.0	28.1		29.7	31.3	
MedDiet during pregnancy (score)	9.34±2.29	10.19±2.48	0.130	9.42±2.17	9.83±2.38	0.175	**9.36±2.39**	**10.65±2.35**	**0.008**	9.77±2.38	9.69±2.41	0.734	10.01±2.59	9.87±2.69	0.643	**9.47±2.56**	**10.61±2.45**	**0.023**	9.66±2.39	10.59±2.43	0.088
Energy intake during pregnancy (kcal/d)	**1892.11 ±468.66**	**2143.82 ±575.45**	**0.016**	**1789.28 ±455.11**	**2049.99 ±584.02**	**0.013**	**1863.54 ±446.98**	**2132.25 ±594.04**	**0.010**	**1709.09 ±375.45**	**2196.97 ±572.95**	**<.001**	**1721.66± 394.41**	**2254.24±582.20**	**<.001**	**1875.24± 452.22**	**2111.13 ±608.79**	**0.030**	**1871.06± 469.60**	**2120.56 ±600.15**	**0.019**
State-trait anxiety inventory score	14.49±7.27	14.37±6.14	0.566	16.02±6.84	13.58±5.92	0.128	15.49±7.62	14.17±6.19	0.550	14.95±7.44	14.04±5.67	0.515	16.07±8.26	13.95±5.60	0.175	15.52±7.63	14.44±6.29	0.586	15.20±7.83	14.21±6.17	0.688

Descriptive statistics are presented with quantitative variables expressed as mean ± standard deviations, and qualitative variables as percentages. P-value for comparisons between categories was calculated by Pearson's chi-square test or one-factor ANOVA tests for categorical variables and continuous variables, respectively. (%). Abbreviations: T, tertile; As, arsenic; Cd, cadmium; MeHg, methylmercury; Pb, lead; PCDD/Fs, polychlorinated dibenzo-p-dioxins and dibenzofurans; DL-PCBs, dioxin-like polychlorinated biphenyls; NDL-PCBs, non-dioxin-like polychlorinated biphenyls; MedDiet, Mediterranean diet; BMI, early pregnancy Body Mass Index; d, day.

Supplementary Table 2. Association of 4-year children psychological problems and tertiles of prenatal dietary toxicants consumption

Emotional and Behavior scale	Dietary toxicants																				
	As			Cd			MeHg			Pb			PCDD/Fs			DL-PCBs			NDL-PCBs		
	T1 <194.83 μg/d	T3 >298.09 μg/d	P	T1 <5.11 μg/d	T3 >6.83 μg/d	P	T1 <3.62 μg/d	T3 >5.51 μg/d	P	T1 <2.92 μg/d	T3 >3.60 μg/d	P	T1 <8.55 TEQ/d	T3 >10.90 TEQ/d	P	T1 <14.00 TEQ/d	T3 >20.55 TEQ/d	P	T1 <133.91 TEQ/d	T3 >201.92 TEQ/d	P
**Syndrome Scales**																					
Emotionally reactive	57.56±9.64	56.77±8.37	0.879	57.03±9.75	57.56±8.78	0.877	57.34±9.39	56.64±8.06	0.878	57.23±10.11	57.95±8.02	0.530	57.41±9.75	56.45±7.51	0.775	57.50±9.82	57.17±8.77	0.874	57.22±9.35	57.16±8.65	0.987
Anxious/depressed	55.88±7.14	56.70±8.00	0.758	56.17±7.96	56.55±7.67	0.805	55.83±6.52	56.06±7.64	0.878	55.63±7.47	57.19±7.78	0.388	55.69±7.18	55.97±6.74	0.719	56.05±7.26	56.27±7.93	0.984	55.72±6.58	56.11±7.64	0.819
Somatic complaints	55.39±6.67	55.50±6.05	0.944	56.73±7.56	54.86±6.12	0.114	55.50±6.80	55.25±6.41	0.971	55.52±6.83	55.91±6.49	0.517	55.33±6.90	55.34±6.49	1.000	55.59±6.92	55.31±6.46	0.915	55.39±6.83	55.09±6.32	0.936
Withdrawn	57.73±6.84	58.58±7.10	0.574	57.84±8.04	57.55±6.79	0.914	57.39±6.94	57.81±7.14	0.793	57.34±7.63	58.19±6.32	0.805	56.86±7.45	57.11±5.78	0.083	57.39±7.08	57.97±7.46	0.840	57.52±6.83	57.91±7.28	0.910
Attention problems	58.64±7.38	58.22±7.16	0.191	58.33±7.07	58.13±7.66	0.455	58.48±7.10	57.34±7.37	0.625	57.42±7.02	58.22±6.97	0.818	58.19±7.30	57.17±7.02	0.720	58.16±6.83	57.81±7.61	0.808	58.22±7.22	57.61±7.42	0.824
Aggressive behavior	55.98±7.21	55.53±7.40	0.814	55.36±6.67	56.36±8.76	0.545	55.98±6.83	54.02±5.37	0.122	55.27±7.07	55.75±6.97	0.932	56.09±8.12	54.28±5.80	0.257	56.28±7.18	54.66±7.02	0.468	55.80±6.76	54.91±7.27	0.705
**Broad-band scales**																					
Internalizing	55.19±11.37	56.02±10.86	0.311	54.84±13.11	55.41±11.33	0.755	54.64±11.65	55.11±11.03	0.935	53.72±12.83	57.09±10.05	0.144	53.63±12.92	54.91±10.06	0.657	54.84±11.96	55.44±11.49	0.739	54.48±11.67	55.34±11.37	0.865
Externalizing	54.11±11.25	54.09±10.36	0.495	53.52±10.91	54.34±12.49	0.620	54.23±10.81	51.73±9.56	0.338	53.20±10.59	54.41±9.97	0.667	53.98±12.06	52.28±9.33	0.610	54.33±11.00	52.42±10.73	0.629	53.98±10.71	52.92±10.53	0.866
Total problems	55.03±11.74	55.06±11.21	0.589	54.73±13.05	55.13±12.90	0.691	54.77±11.36	53.47±10.75	0.756	53.80±12.40	55.80±11.00	0.537	54.39±12.80	53.50±10.08	0.707	54.89±12.02	53.97±11.62	0.913	54.45±11.34	54.03±11.42	0.951
**DSM-Oriented Scales**																					
Depressive problems	56.67±7.01	56.48±6.70	0.961	55.70±6.77	56.95±7.64	0.557	56.16±6.87	55.86±6.42	0.407	55.64±6.86	57.11±6.84	0.485	56.00±7.15	55.63±6.11	0.170	56.52±7.11	56.27±6.91	0.946	55.92±6.81	56.02±6.82	0.360
Anxiety problems	57.30±7.84	58.09±8.31	0.726	57.20±8.56	57.56±8.05	0.956	57.00±7.70	57.33±8.26	0.767	56.92±8.37	58.33±8.24	0.572	57.03±8.00	57.34±7.82	0.798	57.23±8.17	57.58±8.66	0.966	56.75±7.50	57.28±8.44	0.539
Autism spectrum problems	57.48±7.16	58.03±7.03	0.394	57.91±7.77	57.17±6.67	0.664	57.08±7.17	57.34±7.08	0.961	57.48±7.59	57.64±6.55	0.742	57.19±7.65	56.66±6.22	0.570	57.08±7.23	57.39±7.20	0.963	57.22±7.02	57.34±7.21	0.995
Attention-deficit/Hyperactivity problems	58.27±8.17	57.52±7.93	0.299	57.75±7.69	57.70±8.50	0.573	58.42±7.68	55.94±7.21	0.202	56.73±7.23	57.39±7.38	0.761	58.20±8.20	56.13±7.02	0.319	58.53±7.82	56.44±7.80	0.298	58.05±7.78	56.48±7.85	0.540
Oppositional defiant problems	55.23±6.90	55.30±7.22	0.764	55.05±6.85	55.94±7.86	0.301	55.22±6.80	53.89±5.35	0.250	54.97±6.99	55.95±7.33	0.319	55.06±7.27	54.28±6.17	0.528	55.41±7.17	54.42±6.25	0.704	55.00±6.53	54.77±6.58	0.926

P-value for comparisons among categories was calculated by one-factor ANOVA tests for categorical variables, respectively. Abbreviations: T, tertile; As, arsenic; Cd, cadmium; MeHg, methylmercury; Pb, lead; PCDD/Fs, polychlorinated dibenzo-p-dioxins and dibenzofurans; DL-PCBs, dioxin-like polychlorinated biphenyls; NDL-PCBs, non-dioxin-like polychlorinated biphenyls; d, day.
